# *Citrus aurantium* Flowers: Overview of Chemistry, Functionality, and Technological Applications

**DOI:** 10.3390/molecules30040930

**Published:** 2025-02-17

**Authors:** Sepidar Seyyedi-Mansour, Pauline Donn, Paula Barciela, Ana Perez-Vazquez, Rafael Nogueira-Marques, Franklin Chamorro, Maria Carpena, Miguel A. Prieto

**Affiliations:** Universidade de Vigo, Nutrition and Bromatology Group, Department of Analytical Chemistry and Food Science, Instituto de Agroecoloxía e Alimentación (IAA)—CITEXVI, 36310 Vigo, Spain; sepidar.seyyedi@uvigo.es (S.S.-M.); donn.pauline@uvigo.es (P.D.); paula.barciela@uvigo.es (P.B.); ana.perez.vazquez@uvigo.es (A.P.-V.); nogueirarafael29@gmail.com (R.N.-M.); franklin.noel.chamorro@uvigo.es (F.C.)

**Keywords:** biological properties, *Citrus aurantium* L., extraction methods

## Abstract

Bitter orange (*Citrus aurantium* L.), a member of the Rutaceae family, finds global utility in both the treatment of various ailments and its role as a rootstock for *Citrus* species in agriculture. Various parts of *Citrus aurantium* L. have been employed in traditional medicine due to their multifarious therapeutic potential. The blossom of this plant serves as a rich source of bioactive compounds, notably polyphenols, alkaloids, and terpenes. Additionally, it harbors substantial quantities of functional, nutritive, and biologically active compounds, which manifest their presence through antioxidant, antidiabetic, anticancer, antimicrobial, cardiovascular, and neuroprotective properties. The recovery of bioactive compounds is significantly affected by extraction methods. Many conventional methods have been explored for the recovering of bioactive compounds from bitter orange flowers. However, in response to the limitations of conventional techniques, green extraction methods, characterized by their ability to significantly increase the yield and reduce the time, energy, and solvent requirements, have also been assessed for this matrix. Therefore, the study of the functionalities of bitter orange blossoms represents a domain with unexplored research opportunities. Consequently, this review aims to offer a comprehensive insight into the biological properties and medicinal applications of the active compounds found within *C. aurantium*.

## 1. Introduction

Plants have profoundly impacted the human diet throughout history, showcasing notable health-enhancing attributes. Given increasing health concerns and the persistent quest for healthier lifestyles, there has been a surge in interest surrounding naturally derived substances extracted from plants. Citrus stands as one of the paramount fruit trees crops globally, not solely due to its substantial total production, but also owing to its notable economic value [1]. Different parts of the citrus tree hold significance within traditional herbal medicine for addressing diverse health issues. Notably, in Asian herbal practices, the whole unripe and dried fruit is employed to facilitate digestion. In contrast, regions like South America and Mexico use the leaves to cause a sense of tranquility for the mind and soul. Furthermore, the essential oils extracted from the fruits find application in creating liquors and perfumes [2].

Within the array of *Citrus* species, *Citrus aurantium* L. stands out as a distinct variety with versatile applications spanning functional foods, cosmetics, and the pharmaceutical sector. *C. aurantium* L., commonly referred to as sour orange or bitter orange, is an evergreen tree mainly cultivated in tropical and subtropical areas and belongs to the Rutaceae family. *C. aurantium* has garnered recent attention due to its wide range of biological activities. These include anticancer, antibacterial, antioxidant, antidiabetic, and immune-enhancing properties, and potential applications in treating neurological disorders [3]. *C. aurantium* is renowned for its fragrant white flowers, characterized by their delicate nature and arrangement along the shoot axis, occurring singly or in multiple formations [4]. *C. aurantium* flowers have a rich history of usage as a food flavoring agent, contributing to various beverage types and pastries. Beyond culinary applications, these flowers have also found a place in medicinal products due to their attributed antidepressant, anti-infectious, and sedative properties. Moreover, their incorporation into skincare products highlights their versatility across various domains [5]. Many biological properties have been attributed to these flowers, including antioxidant, anticancer, anti-complement, anti-inflammatory, and anti-tumor properties [6,7,8]. Specifically, compounds such as phenolic acids, flavonoids, alkaloids, and terpenoids (essential oils) are potentially responsible for these mentioned properties. They have garnered significant attention owing to their diverse functionalities and inherent potential as nutraceutical agents [9].

Extraction technology plays a fundamental role in influencing the extraction efficiency of phytochemical components and significantly affects their subsequent biological activities. Conventional extraction techniques have been used the most to extract bioactive compounds in citrus. This technique has disadvantages, such as the use of large amounts of solvent; in some cases, it is not environmentally friendly and causes a longer duration of extraction. Consequently, there is an urgent need for a new and efficient extraction approach for materials derived from citrus blossoms. In this context, green extraction techniques have shown promise. For example, Sandhu et al. showed that the ultrasonic method can significantly increase the biological potential of citrus extracts [10]. Also, these techniques are considered more environmentally friendly because smaller amounts of solvent are required, significantly reducing the extraction time. In addition, heat-sensitive compounds are preserved depending on the chosen method and conditions. However, the extraction conditions must be carefully evaluated for the best recovery efficiency. Prior research has commonly employed solvents like methanol, ethanol, acetone, and ethyl acetate, often mixed with water, to extract phenolic and flavonoid substances from plants. The polarity of these solvents is a key consideration in phenolic and flavonoid extraction procedures. Consequently, selecting an appropriate standardized extraction method for these plant compounds is challenging. Due to this, there can be differences in the total levels of phenolics and flavonoids based on the extraction solvents used [11].

The prime objective of this review was to consolidate recent research endeavors pertaining to the bioactive compounds within *C. aurantium* flowers. This entailed a comprehensive exploration of extraction techniques conducive to isolating these compounds, as well as a thorough examination of the biological attributes exhibited by extracts derived from the plant. The aim was to consider the prospective utilization of these extracts as ingredients within the domains of food, pharmaceuticals, and cosmetics. Furthermore, this review serves as a resource for recognizing existing gaps in knowledge, potentially facilitating further investigation into this relatively underexplored species.

## 2. Literature Review Search

The methodology employed for the literature search was structured through different research tools such as Google Scholar, Pubmed, and Science Direct, by finding articles related to the topic, and using a combination of the specific key words, “*Citrus aurantium* L.”, “Bitter orange flowers”, “Biological activities”, “Extraction methods”, and “industrial applications”. The final inclusion was made in accordance with articles and book chapters that were within the scope of the referenced keywords within the last 20 years in order to compile the maximum amount of information, and only appropriate studies were utilized.

## 3. Bioactive Compounds of Citrus Flowers

**Figure 1:**
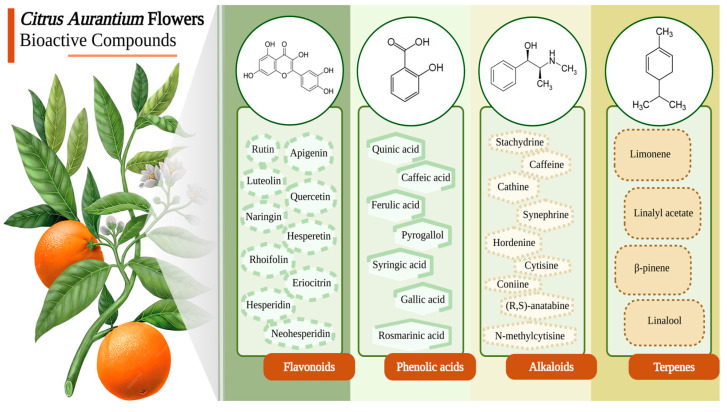
Chemical constituents attributed to *Citrus aurantium* flowers are associated with health-promoting properties. Created in https://BioRender.com (accessed on 20 January 2025).

The *C. aurantium* flower is a rich source of bioactive compounds, which makes it an interesting topic to some authors, and their health-enhancing properties can be attributed to these chemical constituents (Figure 1). The details of each compound within the metabolites of *C. aurantium* flowers are elaborated in the below section.

### 3.1. Phenolic Compounds

As one of the vital chemical components in *C. aurantium*, phenolics have been recognized as having beneficial properties for human health. Polyphenols are a group of secondary metabolites that are widespread in the plant kingdom. They are known to serve various protective functions for plants that are significant to their adaptability and survival. The antioxidant activity of Citrus flowers involves various components, but polyphenols, which comprise flavonoids and phenolic acids, have received considerable attention due to their importance and extensive investigation.

The flowers of *C. aurantium* contain a diverse array of compounds, notably (1) flavonoids, including rutin, apigenin, luteolin, quercetin, naringin, hesperetin, rhoifolin, eriocitrin, hesperidin, and neohesperidin; and (2) phenolic acids, such as quinic acid, caffeic acid, rosmarinic acid, ferulic acid, pyrogallol, syringic acid, and gallic acid. Some authors have reported that *C. aurantium* flowers are a rich source of phenolic compounds, especially flavonoids, which are present in significant quantities [3,5,11,12]. In the *C. aurantium* flower, a noteworthy class of compounds are flavonoids, which can be classified into three categories: flavones, flavanones, and flavanols. Notably, flavones may also be found in the methoxylated form, where most or all hydroxyl groups are methylated. It has been observed that the total amount of flavonoids (TFC) and total phenolics (TPC) present in the *C. aurantium* flowers is 46 ± 2.40 mg/g and 81 ± 1.80 mg/g, respectively [13]. This indicates that the sample under investigation may contain significant quantities of flavonoids, which are natural compounds found in plants and are known for their potential health benefits, including antioxidant and anti-inflammatory effects [14]. According to two studies investigating methanolic *C. aurantium* flower extracts, the phenolic compound pyrogallol exhibited the highest amount, measuring 541 and 526 μg/g in dry weight (DW). In contrast, rosmarinic acid with 75.4 μg/g DW and gallic acid with 212 μg/g DW had a lower amount [11,12]. Moreover, it has been found that 5-hydroxy-6,7,3′,4′-tetramethoxyflavone (HTF) is present in the flowers of *Citrus aurantium* L. var. *amara* Engl (*C. aurantium* var. *amara*) and exhibited remarkable antioxidant and anti-tumor properties [15]. Tannins, a group of polyphenolic biomolecules, have been identified as existing within the flowers, leaves, and peels of *C. aurantium* L. These compounds play a crucial role in protecting against predation and regulating plant growth. These molecules play a crucial role in defending the plant against predation while also serving as a regulator for its growth [16]. Taken together, these findings underscore the influence of chemical structures on the interaction with solvents, consequently influencing the extraction yield of compounds.

### 3.2. Other Compounds

*C. aurantium* flowers are a veritable source of active metabolites, comprising various constituents beyond phenolic compounds. Some research has reported the detection of various bioactive compounds, including alkaloids, essential oil, polyterpenes, and sterols, within samples of *C. aurantium* flower extracts [9,16]. Alkaloids with nitrogen atoms constitute the largest category of naturally occurring compounds identified in *C. aurantium*. Every single alkaloid found in bitter orange contributes to an elevation in the metabolic rate as well as an increase in the rate of fat oxidation [2]. A total of nine alkaloid compounds were successfully identified within the flower of *Citrus aurantium* L. var. *amara* Engl. (CAVA) through the employment of an LC–MS assay. Among these compounds, stachydrine, caffeine, and cathine emerged as prominent alkaloids. It has been substantiated that this flower holds considerable potential for inducing anti-obesity effects and regulated lipid metabolism in steatosis Hep G2 cells. Synephrine, hordenine, cytisine, N-methylcytisine, coniine, and (R, S)-anatabine are additional compounds that were identified in this study. Across various studies, these compounds have exhibited a range of activities, including the inhibition of glucose production, cardiovascular benefits, and protective properties against lung damage [17]. Citrus essential oils are generally acknowledged as safe (GRAS), owing to their extensive range of biological activities. Besides the activities mentioned, undiluted essential oils are traded at considerable prices within the international market, finding significant demand in the perfumery sector and the cosmetic industry. Based on the findings of one study, it has been observed that the essential oil extracted from *C. aurantium* flowers is composed of a complex combination of terpenes, terpenoids, aliphatic and aromatic hydrocarbons, sulfur-containing compounds, phenolics, polyhydric alcohols, and other oxygenated phytochemicals [18]. The main component in the essential oil of *C. aurantium* flowers is linalool, followed by d-limonene, linalyl acetate, and β-pinene [19,20,21]. However, according to the findings of [22], it has been observed that the essential oil derived from the flowers of *C. aurantium* in Tunisia primarily comprises limonene as its principal constituent. Limonene, a prevalent terpene compound widely found in nature, constitutes a significant proportion of various essential oil compositions. The variations observed in the composition of essential oils can be attributed to multiple factors encompassing environmental aspects (such as geography, climate, and soil conditions), plant parts, collection time, genetic influences, and disparities in the extraction techniques employed. Similar findings were reported in the study by Liang et al., where oxygenated monoterpenes were identified as the primary constituents of the essential oil from *Citrus aurantium* flowers, followed by monoterpene hydrocarbons. The major components include linalool, linalyl acetate, and limonene. Pharmacological studies have shown that the oral and intraperitoneal administration of *C. aurantium* essential oil reduces sleep latency, prolongs sleep duration, and decreases locomotor activity in mice. Additionally, GC-MS analysis has identified several key volatile compounds, including α-pinene, 3-carene, 1,2-oxolinalool, hotrienol, sabinene, terpinolene, and geraniol [20].

## 4. Biological Activities of Citrus Flowers

### 4.1. Antioxidant Activity of Citrus aurantium Flowers

The antioxidant activity of a plant extract is the capacity of the extract to delay or prevent the oxidation of free radicals and thus limit the harmful consequences of oxidation. Nowadays, migration from synthetic to natural antioxidants is increasing due to health reasons. Foods, pharmaceuticals, and cosmetics industries are looking for new natural sources of ingredients with antioxidant properties for health and environmental purposes. In the literature, the antioxidant activity of the extracts of *Citrus aurantium* flowers (*CAF*) has been reported by many authors (Table 1). For example, in the study conducted by Değirmenci and Erkurt, 2020, the radical scavenging activities of hydrogen peroxide (H_2_O_2_) and 1,1-diphenyl-2-picrylhydrazyl (DPPH) of an ethanolic extract of *CAF* were evaluated. From their findings, it was found that this extract presents antioxidant properties, as seen from the results of the half inhibitory concentrations (IC_50_) that were 66.50 ± 2.70 and 96.07 ± 1.26 µg/mL, respectively, for H_2_O_2_ and DPPH free radical scavenging activities. They also determined the total phenolic content (TPC) and total flavonoids content (TFC) of the ethanolic extract of *CAF*, estimated at 81.37 ± 3.2 mg gallic acid equivalent (GAE)/g of extract (E) and 20.34 ± 2.68 mg of quercetin equivalent (QE)/g E, thus correlating with their antioxidant activity [9]. Other authors [11] have studied the antioxidant activity of *CAF* by determining the ferric reducing antioxidant power (FRAP) and DPPH radical scavenging of three different extracts of the *Citrus aurantium* flower: an ethanolic extract (EE), a methanolic extract (ME), and an infusion extract with boiled water (IE). Their results showed that the ME presented higher DPPH free radical scavenging activity and ferric reductive antioxidant power in comparison to the other extract; meanwhile, it still had a smaller concentration of 300 µg/mL of BHT or α-tocopherol. The results presented as the percentage of antioxidant power at 300 µg/mL of the ME, EE, and IE of CAF were as follows for the DPPH radical scavenging activity: 55.32%, 52.41%, and 50.46%; and for FRAP: 51.70%, 47.60%, and 43.50%. Similarly, the evaluated TPC and TFC of the tree extract followed the same behavior, with the ME presenting higher values (TPC: 4.83 ± 0.05 mg GAE/g DW, TFC: 4.11 ± 0.05 mg rutin equivalent (RE)/g DW) followed by the EE (TPC: 4.55 ± 0.01 mg GAE/g DW, TFC: 3.83 ± 0.05 mg RE/g DW) and the IE (TPC: 3.93 ± 0.58 mg GAE/g DW, TFC: 1.88 ± 0.01 mg RE/g DW). The variability of results amongst different authors could be due to the difference in extraction methods, the type of solvent, and the origin and treatment method of the sample.

### 4.2. Anticancer Properties of Citrus aurantium Flowers

The management of cancer with compounds or extracts from natural sources is a recent and efficient treatment method that, additionally, has been shown to exert a lesser secondary effect. Through their antioxidant activities, phenolic compounds in general and particularly flavonoids, identified in plant extracts like *CAF*, can play the role of antiproliferative agents to avoid or slow down the apoptosis of cancer cells [24]. In this way, the anticancer potential of *CAF* extracts has been studied by many authors; some of the results obtained are shown in Table 1. For example, the effects of the methanolic extract of *CAF* were evaluated on Chang liver cancer cells, breast cancer cells (MCF-7, MDA-MB 231), and colon cancer cells (HT-29). These authors found that the cytotoxicity of the extract was more efficient on MDA-MB 231 with an IC_50_ of 49.74 ± 0.75 µg/mL [11]. Also, other authors have found that limonexic acid (LA) and 5-hydroxy-6,7,3′,4′-tetramethoxyflavone (HTF), two bioactive compounds isolated from *CAF*, present a concentration-dependent inhibitory effect on the proliferation of a human skin cancer model (B16) and a hepatocellular carcinoma cell line (SMCC-7721). They show that HTF presents greater inhibitory activity on SMCC-7721 cell growth, with a value of 88.20% for an extract concentration of 200 µg/mL. They concluded that HTF has a higher inhibition effect on the morphological variation and proliferation of the tested cancer cells compared to the control ‘5-Fluorouracil’ [5,15]. Additionally, polysaccharides extracted from *Citrus aurantium* have been studied for their anticancer activity with plausible results, showing their high potential in the treatment of cancer [25]. Also, limonene, a compound present in the essential oil of *CAF*, has proven, through a test on animals, that it presents a positive response against cancers of the liver, pulmonary system, breast, skin, colon, and pancreas by slowing down the growth of their development [26]. Thus, the presented results of the different studies conducted on this plant lead to the conclusion that it is a very good source of anticancer compounds for further application in pharmacology as a natural ingredient.

### 4.3. Antimicrobial Activity Citrus aurantium Flowers

Pathogenic microorganisms are hazards that could be very harmful to human beings, especially above a certain dose once ingested. In nature, there are plant extracts or compounds extracted from plants that can fight or inhibit the activities/proliferation of these pathogenic microorganisms. The capacity of a plant extract to play this role is characterized by its antimicrobial activity. Thus, the plant extracts of *CAF* as well as their essential oil extracts (EO) have been studied by many authors for this purpose, as seen in Table 1. This is the case of the study conducted by Dhekra Trabelsi and their collaborators, which analyzed the in vitro antimicrobial activity of the EOs and methanolic extracts (MEs) of *CAF* against the yeast culture *Candida albicans*, five Gram-negative bacteria (*Klebsiella pneumoniae cefotaxime resistant*, *Klebsiella pneumoniae* ATCC 1388, *Salmonella typhimurium*, *Pseudomonas aeruginosa* ATCC 27853 (CIP 76110), and *Escherichia coli* ATCC 25922 (CIP 7624)), and three Gram-positive ones (Methicillin-resistant *Staphylococcus aureus*, *Staphylococcus aureus* ATCC 25923 (CIP7625), and *Listeria monocytogenes* ATCC 19111). They found that the MEs of *CAF* only inhibited the development of *Staphylococcus aureus* ATCC 25923 with 1.2 mm as inhibition diameter, while the EO of CAF presented antimicrobial activity against all the tested microorganisms with a minimal inhibition concentration (MIC) of 75 µg/mL for most of them, except *Staphylococcus aureus* ATTC (˂75 µg/mL) and *Listeria monocytogenes* ATTC (500 µg/mL) [16]. Moreover, in the research conducted by Degirmenci and Erkurt, 2020, three different studied extracts of CAF (water, methanol, and ethyl acetate, respectively, WE, ME, EE) presented effective antimicrobial activity against five foodborne pathogens in rice pudding (*Escherichia coli* O157:H7, *Staphylococcus Typhimurium Staphylococcus aureus*, *Bacillus cereus*, and *Listeria monocytogenes*), with the ME being the best. They found that the MIC was between 781 and 6250 mg/L for the WE, 390 and 3124 mg/L for the ME, and 6250 and 12,500 mg/L for the EE; while the minimal bactericidal concentration (MBC) was between 1562 and 12,500 mg/L for the WE, 390 and 6250 for the ME, and 12,500 and 25,000 mg/L for the EE [14]. These authors concluded that the ME of *CAF* or isolated bioactive compounds of this extract are possible sources of natural ingredients for application as natural preservatives in food and pharmaceutical industries.

### 4.4. Anti-Inflammatory Activity of Citrus aurantium Flowers

Inflammation is defined as the body’s immune response to infections, injury, or metabolic stress [27]. This phenomenon can be harmful to human beings and needs to be regulated through anti-inflammatory compounds. Thus, some flavonoid-rich plant extracts have demonstrated anti-inflammatory activity. That is the case of the *CAF* extract that was studied by Karimi and collaborators. These authors found that the ME of *CAF* at different concentrations was able to inhibit induced nitric oxide (NO) production (by IFN-γ and LPS) from the macrophage RAW 264.7 cells. They evaluated the NO production at 14.40 µM while applying the ME at a concentration of 100 µg/mL. Also, they found that even at the highest concentration of the extract amongst the studied ones (100 µg/mL), the RAW 264.7 cell viability was still higher than 90%, showing the safety of the tested *CAF* methanolic extract [11].

### 4.5. Anti-Obesity Activity of Citrus aurantium Flowers

Obesity, referring to an excessive accumulation of fat, is a metabolic disorder generally associated with diabetes and hypertension. Some plants of traditional pharmacopeia have been suggested as a natural treatment for obesity-associated diseases with the advantage of presenting fewer adverse effects [28,29]. Some authors have studied the anti-obesity activity of *CAF* extract through its ability to inhibit lipid accumulation. Thus, in the study carried out by Li et al., (2021) in vitro and in vivo screening of the anti-obesity activity of the chloroform (CHL) extracts of *CAF* were evaluated. These authors found that this extract is not only able to decrease the metabolic syndrome of high-fat-diet mice, but in addition, can inhibit the differentiation of 3T3-L1 preadipocytes, a key factor of excessive energy storage [29,30]. These results support the reason why they have been used for many years in traditional medicine to manage disorders related to obesity. However, the authors suggested that additional research regarding the bioavailability and toxicity of this extract needs to be carried out [29].

### 4.6. Anti-Amnesic Activity of Citrus aurantium Flowers

Alzheimer’s disease (AD), known as a progressive neurodegenerative disorder that mainly affects the older population, is associated with a loss in memory and cognitive impairments [31]. In vivo studies of the anti-amnesic potential of plant extracts on scopolamine-induced memory impairments have been studied by Rahnama et al., (2015) in order to evaluate capacity for memory and learning impairments to be reduced. Their results show the significant efficiency of the tested *CAF* extracts in passive avoidance response tests. Also, the results of a Morris water maze test indicate that during trial sessions, this extract can reduce escape latency. Concerning the effect of *CAF* extract on malondialdehyde (MDA) serum, this extract significantly reduces MDA serum levels. Finally, these authors concluded that there is a beneficial effect of the ME of *CAF* in the treatment of Alzheimer’s disease and the possibility for application of the extracted bioactive compounds of *CAF* in pharmaceutical industries [32].

## 5. Extraction Techniques of Bioactive Compounds of Citrus Flower

In recent decades, there has been an increasing interest in obtaining several compounds (e.g., phenolic compounds, tannins, and flavonoids) naturally present in different matrixes (e.g., plants and seaweed) due to their biological activity. In this way, the extraction step is an essential feature, so that the compounds previously mentioned can be released from the cellular medium [33]. Therefore, extraction is defined as the process in which the cell is disrupted to liberate the compounds inside it. Extraction techniques can be classified into two groups: conventional extraction techniques, in which maceration, percolation, digestion, and Soxhlet extraction are included [33]; and new extraction techniques, in which ultrasound- assisted extraction, pressurized liquid extraction, ultrasound-assisted extraction, enzyme-assisted extraction, and supercritical fluid extraction are included [33]. In this section, the studies conducted regarding *C. aurantium* flowers to this date using either conventional or new extraction techniques are compiled. Moreover, the most relevant results are included in Table 2.

### 5.1. Conventional Extraction Techniques

Conventional extraction techniques have been the most studied techniques applied in *C. aurantium* flowers. Zhu et al. (2021) extracted the essential oils (EOs) of *C. aurantium* L. var *amara* flowers by applying mechanochemical-assisted extraction. Mechanochemistry is a process where the mechanical energy produced by applying force (e.g., shear, friction, or impact force) in solid matrixes leads to changes produced by the substance’s internal energy [34]. Flowers were pretreated by applying ball milling for several minutes until a powder was achieved. Then, the extraction was set using n-hexane as an extraction solvent for 30 min at room temperature. Since no heat is applied, the milling step is crucial to obtain high recovery yields. Hydrodistillation and reflux extraction were also performed as a control. For the hydrodistillation extraction, water at 140 °C was used as a solvent for 6 h, while reflux extraction was performed with n-hexane at 70 °C for 4 h. The recovery yields of the EO obtained using mechanochemical-assisted extraction, reflux, and hydrodistillation were 6.60%, 2.08%, and 0.25%, respectively. Thus, by applying MCAE, Zhu et al. (2021) achieved a recovery yield 26 times higher than hydrodistillation and three times higher than reflux extraction. Moreover, MCAE was able to obtain higher yields in less time and without applying heat, which may be an advantage for the stability of the thermolabile compounds extracted. Hydrodistillation was also applied by Sidi et al. to extract the EO from *C. aurantium* flowers [35]. Hydrodistillation is a traditional extraction methodology usually applied to obtain the EO from plant materials [38]. It can be performed in different ways: water distillation, water and steam distillation, and direct steam distillation [39]. Different authors have studied hydrodistillation applied in flowers from *C. aurantium*, especially for EO extraction. For EO extraction, water at 100 °C for 360 min was employed, obtaining 0.31% of the extraction yield, with limonene being the main compound of the EO. Moreover, the antimicrobial activity against *E. aerogenes*, *S. typhi*, *M. luteus*, *K. pneumoniae*, *E. coli*, *B. subtilis*, and *S. aureus* was also studied, showing positive results against all these pathogens [35]. Degirmenci and Erkurt, 2020, also obtained positive antimicrobial and antioxidant activity results from EOs extracted from *C. aurantium* flowers by applying steam distillation for 4 h and obtaining 0.57% yield recovery. In this study, Soxhlet extraction was also applied using ethanol for 6–8 h, obtaining 16.38% of the EO and showing the suitability of this process [9]. In another study run by J. Li et al., steam distillation was applied in *C. aurantium* flowers to obtain the EO. The results show a high content in flavanones (17.93% of the EO extracted), showing great anti-hyperlipidemia activity [37]. Continuing with Soxhlet extraction, this is a traditional extraction technique that has been already used in flowers of *C. aurantium.* It is identified as the standard methodology to which new extraction techniques are compared. In this way, Soxhlet extraction combines both maceration and percolation extraction techniques since the solvent reaches the boiling point and the condensed drops pass through the solid matrix in a process that takes almost 8 h [40]. Degirmenci and Erkurt, 2020, ran a study in which this technique was applied in *C. aurantium* flowers using four different solvents (water, methanol, ethyl acetate, and hexane) in their respective boiling points for 8 h. In this way, a comparison of the different solvent suitabilities was made, where methanol showed the highest recovery with 17.46%, followed by water with 15.52%. Moreover, the authors studied the antioxidant and antimicrobial activity of the extracts against different pathogens in rice pudding, showing positive results and suggesting their potential application as shelf-life extenders in dairy desserts [14]. Percolation is also a traditional extraction technique that consists of passing a solvent through a solid material drop by drop. The flow ratio of the extracted solvents is 5 mL/min when 1 kg of plant material is used [33]. Hydrodistillation and percolation extraction were used for the phenolic, flavonoid, and tannin determination and EO recovery from *C. aurantium* blossoms, respectively. Methanol was used for percolation extraction to determine the phenolic, flavonoid, and tannin content, while water at 100 °C for 4 h was applied for EO extraction. The authors determined 26 volatile compounds in the EO, with linalool being the most abundant (25.70%) of the total EOs extracted (which was not specified in the work). Moreover, the total phenolic compounds, the total flavonoids, and the tannins present in the extract were quantified, being 8.78 mg GAE/g, 4.86 mg EC/g, and 0.06 mg EC/g, respectively. In this way, the authors determined the free radical scavenging activity of the methanolic extracts, showing an IC_50_ of 20 µL/g [36]. Solvent extraction is a process where the transfer of compounds takes place by putting the solvents in contact with different solubility or distribution coefficients [41]. This extraction technique was applied to *C. aurantium* flowers with no petals. Two runs were carried out for 30 min: one using methanol as extraction solvent, and the other using ethanol 50%, being 5.10% and 8.60% of the extraction recovery obtained, respectively. Moreover, the extracts showed a potential tyrosinase inhibition, and antioxidative, antibacterial, and anti-wrinkle activity, with potential application in the cosmetic industry [12].

Considering the data shown, traditional extraction techniques have been applied in *C. aurantium* flowers to extract different compounds, mainly EOs. In this way, higher recovery yields were obtained with Soxhlet extraction. However, this methodology needs long extraction times and high quantity solvents, so alternative pathways should be considered since the application of this EO in both the food and pharmacology industries could be interesting.

### 5.2. New Extraction Techniques

New extraction techniques have been slightly studied using flowers from *C. aurantium* as the matrix. In this way, a study compared both traditional and new extraction methodologies for EO recovery. The new methodologies were solvent-free microwave extraction (SFME), solventless microwave extraction (SLME), and ohmic-assisted hydrodistillation (OAHM). Water was used as the extraction solvent in both SLME and OAHM, and the extraction time was 50 min for all the treatments. The highest extraction yield of the three new methodologies was obtained when SLME was applied, achieving 0.21%. For SFME and OAHM the extraction yields were 0.17 and 0.05%, respectively. Moreover, the results of the EO showed how SLME caused the selective extraction of linalool, which was also the main compound of all the EOs obtained. Finally, in this study, it was shown that the extraction technique can affect the yields of the compounds extracted, with limonene and b-pinene showing significant differences [4]. Microwave-assisted extraction was also applied in *C. aurantium* flowers by Almalki, W.H. In this study, a mixture of water and dichloromethane at 25 °C were used as the extraction solvents for 25 min, achieving 1.19% EO. Moreover, the antioxidant, anti-inflammatory, and antiapoptotic activity, as well as the protection against hepatocellular I/R-induced damage, was studied, showing positive results [18]. Another study, in which ultrasound-assisted extraction was applied for the EO recovery of *C. aurantium* flowers, showed the anti-hyperlipidemia activity of the extract obtained using ethanol 80% for 30 min and 100 Hz [37].

As well as with traditional extraction techniques, new extraction methodologies applied in *C. aurantium* flowers are conducted for EO recovery. However, the studies using novel extraction techniques applied in this matrix are limited. Moreover, the extraction yields obtained are lower than those obtained with Soxhlet extraction, so further research is needed, especially considering the current global concern to apply less toxic and more environmentally friendly procedures during industrial processes. Table 3 summarizes the benefits and drawbacks of the different conventional and advanced extraction methods.

## 6. Potential Applications in Food, Pharmaceutical, and Cosmetic Industries

**Figure 2:**
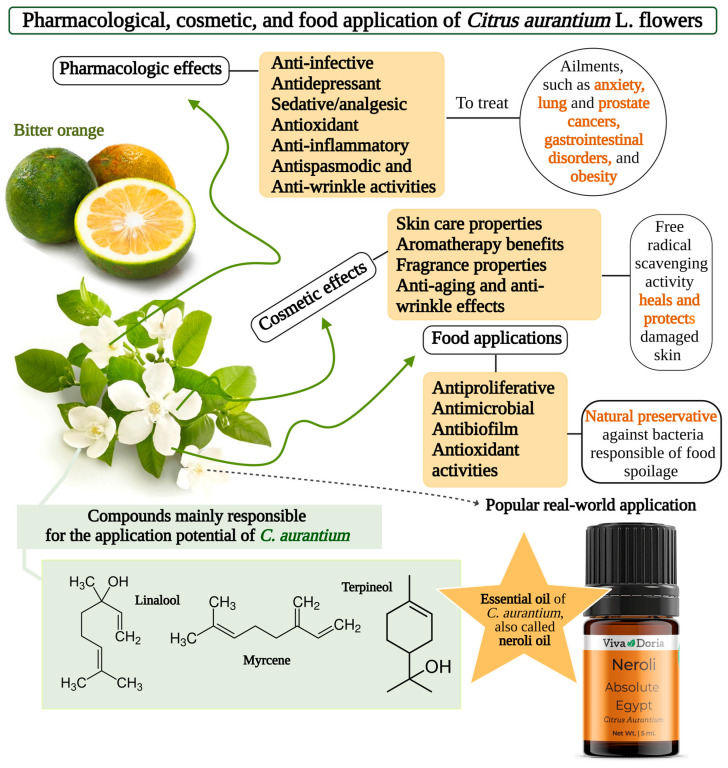
An overview of the different applications of the *Citrus aurantium* flower. Created in https://BioRender.com (accessed on 20 January 2025).

*C. aurantium* flowers have a widespread application as a flavoring agent in several food products, such as dairy products, or in the pharmaceutical and cosmetic industries as an ingredient in many anti-infective, antidepressant, sedative, and even skin care products, as is shown in Figure 2 [5,9].

A popular use is the essential oil of *C. aurantium*, also called neroli oil, utilized in aromatherapy for its central nervous system stimulation, blood pressure reduction, sedative, analgesic, anti-inflammatory, antispasmodic, digestive, and diuretic properties [51]. These properties are based on the chemical composition of the species; in fact, among the most prominent active compounds that compose *C. aurantium*, pectin and hesperidin represent a large proportion, which has meant a significant increase of development and application [52]. In addition, D-limonene, closely followed by several other monoterpenes such as linalool, β-myrcene, and α-terpineol, are often predominant compounds in *C. aurantium* [53]. Its composition also excels in many other phytochemicals; for example, in a study conducted to demonstrate the protective effects of the essential oil (EO) of *C. aurantium* flowers on liver injury, the results revealed that the oil had a high concentration of terpenes, along with their oxygenated derivatives, hydrocarbons (aliphatic and aromatic), phenolics, alcohols, aldehydes, and other essential oxygenated phytochemicals, which caused rats to produce fewer apoptotic cells than rats exposed to ischemia accompanied by reperfusion without the administration of the EO. *C. aurantium* EO was proven to have antioxidant and anti-inflammatory effects [18]. The antimicrobial properties of the EO are also remarkable, with demonstrated effects against Gram-negative and Gram-positive strains and against opportunistic pathogenic bacteria, which have a crucial role in food-borne infections or small skin wounds [54].

In a separate study, they were able to demonstrate the potential of *C. aurantium* flower extract in its application as a natural preservative against bacteria responsible for food spoilage to extend the shelf life of dairy desserts. In the study, the phytochemical composition and the in vitro antioxidant and antibacterial activity of different extracts were ascertained. The methanolic extract had a total phenolic content (87.96 mg GAE/g) and flavonoids (28.20 mg QE/g) that directly correlated with the antioxidant activity that showed an IC_50_ value of 87.15 μg/mL for DPPH. Moreover, the extracts were effective, showing their inhibitory action on all the tested bacterial species and highlighting a minimum bactericidal concentration (MIC) of 390 μg/mL for *Staphylococcus aureus*. In addition, the presence of bioactive agents such as polyphenols, flavonoids, alkaloids, and terpenes was identified, which is key for further use in the food industry [14]. In the field of pharmaceutical and cosmetic application but also based on the potential for bitter orange blossom to be used as an antibacterial, Benzaid et al. analyzed the effects of *C. aurantium* EO on the oral bacterium *Streptococcus mutans* and evaluated its toxicity in contact with gingival epithelial cells. The results showed a decrease in the growth of *S. mutans* (OD_600_ decreased to 0.09 compared to the control (0.24) with 0.30 µg/mL of the EO in 24 h) and the degradation of its mature biofilms (equivalent or superior to the effect obtained with gentamicin at 3 µg/mL), in addition to a marked decrease in certain virulence genes (*comC*, *comD*, *comE*, *gtfB*, and *gtfC*). This study proved that, even at low concentrations, the EO of *C. aurantium* is effective in the reduction in bacterial outgrowth and the degradation of biofilms, which constitutes an interesting approach in the design of mouth rinses [55].

The study examined the impact of *Citrus aurantium* L. flower extract on primary dysmenorrhea in young women through a double-blind, randomized controlled trial. Participants received either the extract, mefenamic acid, or a placebo for three days at the beginning of menstruation. The extract significantly reduced pain severity, outperforming mefenamic acid on the first and second days. It also demonstrated a favorable safety profile with minimal side effects. The therapeutic effects are attributed to its bioactive compounds, including flavonoids (linalool, hesperidin, and naringin), polyphenols, and limonoids, which exhibit anti-inflammatory, antioxidant, and antispasmodic properties. These findings suggest *C. aurantium* is a promising natural remedy for menstrual pain management [43].

Applying it also to the cosmetic industry, Chen et al., (2022), tested the anti-aging and anti-wrinkle effects of *C. aurantium* flowers by studying their toxic and tyrosinase inhibitory effects on human epidermal melanoma cells (HEMn) along with their melanin content. The 50% fermented ethanolic extract exhibited remarkable antityrosinase, antioxidant, antibacterial, and anti-wrinkle activity (largely attributed to the presence of neohesperidin, which can reduce the activity of the MMP-1 enzyme). They also reported that it may be considered safe as it exerts no toxic effect on cells of the normal human skin fibroblast line CCD-966 and that it is capable of reducing melanin formation [12].

On the other hand, Pasandideh et al., (2021) screened the antioxidant and inhibitory effects of *C. aurantium* petal extract on acetylcholinesterase and amyloid nanobiofibril production from bovine serum albumin (BSA). The phytochemical screen identified chemical compounds including carbohydrates, phytosterol, saponins, tannins, proteins, essential oils, terpenoids, and flavonoids. D-limonene and daphnetin were the predominant antioxidant compounds in the extracts, with 9.53% and 5.54%, respectively. The antioxidant capacity assessed by a DPPH assay was 8 mg/mL at an extract rate of 94.25% (2.36 mg/mL EC_50_) and the extract reduced acetylcholinesterase activity by less than 47.04% (42.80 mg/mL IC_50_). The results are supportive of the application of *C. aurantium* petals in its use as a natural product to decrease Alzheimer’s disease complications [56]. Due to the richness of secondary metabolites in *C. aurantium*, it has been used to treat several other pathologies or ailments, such as anxiety, lung and prostate cancers, gastrointestinal disorders, and obesity [25]. For instance, the benefit of lavender and *C. aurantium* EO in the recovery of anxiety and nervousness in patients has been reported in conscious ICU patients [57].

Sweet orange (*Citrus sinensis*) and sour orange (*Citrus aurantium*) are globally popular and marketable citrus varieties. They account for 50% of world citrus production, with 73 million metric tons per year [58]. By employing both species (*Citrus sinensis* and *C. aurantium*), Sevindik et al. determined their chemical components and antiproliferative activities with the aim of assessing their use in the cosmetic and pharmaceutical industry as natural perfume, as well as anticancer agents. The results pointed out compounds such as nerolidol (28.07%), 2,6,10-dodecatriene-1-ol, (15.11%), and linalool (14.47%) in the EO of *C. aurantium*, and nerolidol (22.13%), linalool (14.06%), and sabinene (10.83%) were identified in the EO of *C. sinensis*. These findings support that these two EOs exhibit a strong antiproliferative effect along with low cytotoxicity values at low and medium concentrations. Nevertheless, they caused higher apoptotic deformation of the cellosaurus cell line (FL), which suggests the necessity of investigating the cytotoxic effect [59].

By way of discussion, after compiling the available bibliography, it is seen that the applications taken from the flower of the species *C. auranitum* are scarce, despite its potential and abundant chemical composition in active compounds. In the preceding sections, we have described current studies in which the capacity of this species of bitter orange has been tested with the objective of real applications in the cosmetic, pharmaceutical, and food industries.

## 7. Conclusions

The scientific evidence confirmed the benefits of medicinal plants, particularly flowers, and underscored their potential as valuable resources for wide-ranging functions and health advantages. However, certain species have not undergone comprehensive research regarding their chemical composition and biological efficacy. The analysis of *C. aurantium* flowers has exposed their extraordinary chemical composition. Regarding obtaining a more thorough understanding of their scientific properties, this present review has outlined the various bioactive substances discovered in *C. aurantium*, focusing on the phenolic, alkaloid, and terpene compounds that have been documented. Also, eco-friendly and sustainable extraction techniques have been summarized to optimize the yield and preserve the bioactive compounds of *C. aurantium* flowers. Methods such as supercritical fluid extraction, ultrasound-assisted extraction, and microwave-assisted extraction have demonstrated great potential in improving the efficiency and environmental sustainability of the extraction process. These advanced techniques not only enhance the quality of the extracted compounds but also align with the increasing demand for green and sustainable practices in the industry. To sum up, the flowers of Citrus aurantium are a valuable and flexible resource with substantial potential in diverse industries such as the food, nutraceutical, and pharmaceutical industries. It is imperative to continue research and development efforts to fully exploit their capabilities, enabling the development of innovative applications that make use of their chemical diversity and biological functions, thereby promoting sustainability and economic progress.

## Figures and Tables

**Table 1:** Biological activities of *Citrus aurantium* L. extracts.

Solvent	Activity															Ref.
**Antioxidant Activity**																
	EY	TPC	TFC	DPPH			H_2_O_2_			NO			Fe^2+^			
	*%*	mg *GAE*/g *DW*	mg *QE*/g *DW*	*IC*_50_ (µg/mL)			*IC*_50_ (µg/mL)			*at* 800 µg/mL			*at* 800 µg/mL			
H_2_O	15.52	71.45	19.02	191.08			142.86			-			-			[9]
EtOH	17.46	87.96	28.20	87.15			54.96			-			-			
EA	4.63	40.48	10.42	707.88			465.76			-			-			
Hexane	3.14	27.12	12.61	809.19			674.56			-			-			
EtOH	-	4.55	3.83	52.41% *			47.60% *			-			-			[11]
H_2_O	-	3.93	1.88	50.46% *			43.50% *			-			-			
MeOH	-	4.83	4.11	55.32% *			51.70% *			-			-			
EtOH	12.00	78.76	12.11	723.10			902.30			30.00%			6.30%			[23]
**Antimicrobial Activity**																
	*E. coli*	*S. typhimurium*	*S. aureus*	*MRSA*		*B. cereus*		*L. monocytogenes*		*P. aeruginosa*		*K. pneumoniae*		*C. albicans*		
EO (MIC, µg/mL)	75	75	<75	75				500		75		75		75		[16]
H_2_O (MIC, mg/mL)	6250	6250	781	-		1562		781		-		-		-		[14]
H_2_O (MBC, mg/mL)	12,500	12,500	1562	-		1562		1562		-		-		-		
MeOH (MIC, mg/mL)	3124	3124	390	-		390		1562		-		-		-		
MeOH (MBC, mg/mL)	6250	6250	390	-		781		1562		-		-		-		
EA (MIC, mg/mL)	12,500	12,500	12,500	-		6250		6250		-		-		-		
EA (MBC, mg/mL)	25,000	25,000	12,500	-		12,500		12,500		-		-		-		
**Anticancer Activity**																
MeOH	I**C_50_** μg/mL: Chang liver (>); MCF-7 (152.34); MDA-MB 231(49.74); HT-29 (96.23)															[11]
HTF	Inhibition rate: SMCC-7721 (88.20% at 200 µg/mL); Hela cells (71.00% at 200 µg/mL); B16 (54.20% at 50 µg/mL)															[15]
LA	Inhibition rate: SMCC-7721 (50.30% at 200 µg/mL); Hela cells (42.80% at 200 µg/mL); B16 (39.60% at 50 µg/mL)															

Abbreviations: EY: extraction yield; TPC: total phenolic content; TFC: total flavonoid content; DPPH: 2,2-Diphenyl-1-picrylhydrazyl radical scavenging; H_2_O_2_: hydrogen peroxide scavenging activity; NO: nitric oxide scavenging activity; Fe^2+^: ferric chelating ability; FRAP: ferric reducing antioxidant power; IC50: half inhibitory concentration; MIC: minimum inhibitory concentration; MBC: minimum bactericidal concentration, EtOH: ethanol; EO: essential oil; H_2_O: water; EA: ethyl acetate; MeOH: methanol; HTF: 5-hydroxy-6,7,3′,4′-tetramethoxyflavone; LA: limonexic acid. Note: results with (*) for DPPH and H_2_O_2_ were tested at 300 µg/mL.

**Table 2:** Comparison between conventional and new extraction techniques to obtain compounds from *Citrus aurantium* flowers.

Extraction Technique	Operational Conditions						Pre-Treat.	Form	Compound	Yield (%)	Activity	Application	Ref.
	*S*	*T* (°C)	*P* (bar)	*t* (min)	*F* (Hz)	*Pw* (W)							
**Conventional Extraction Techniques**													
Mechano-chemical	n-hexane 1:5 (*w*/*v*)	RT	ns	30	ns	ns	Ball milling	Powder	EO	6.60	ns	ns	[34]
Reflux extraction	n-hexane 1:8 (*w*/*v*)	70	ns	240	ns	ns	Grinding			2.08	ns	ns	
Hydro-distillation	H_2_O 1:20 (*w*/*v*)	140	ns	360	ns	ns	Grinding			0.25	ns	ns	
Hydro-distillation	H_2_O	100	ns	360	ns	ns	Dried	ns	EO	0.31	Antimicrobial activity against *E. aerogenes*, *S. typhi*, *M. luteus*, *K. pneumoniae*, *E. coli*, *B. subtilis*, *S. aureus*	ns	[35]
									Limonene	40.81			
									Linalool	26.66			
									ᵞ-elemene	7.97			
									α-terpineol	4.97			
									α-terpynil acetate	2.07			
Hydro-distillation	H_2_Od	100	ns	240	ns	ns	Crushed	ns	Linalool	25.7	ns	ns	[36]
Percolation	MeOH	RT	ns	ns	ns	ns	Dried	Powder	TPC	8.78 mg EAG/g DW	Scavenging activity	ns	
									TFC	4.86 mg EC/g DW			
									Tannin	0.06 mg EC/g DW			
Solvent extraction	MeOH	ns	ns	30	ns	ns	Grinding	Powder	Extract	5.10	Tyrosinase inhibition, antioxidative, anti-wrinkle treatments	Cosmetic: skin whiteners and anti-wrinkle treatments	[12]
	EtOH 50%									8.60			
Soxhlet extraction	H_2_O	BTB	ns	360–480	ns	ns	Grinding	Powder	Extract	15.52	Antioxidant and antimicrobial	Natural preservative against food spoilage in dairy desserts	[14]
	MeOH									17.46			
	EtOAc									4.63			
	Hexane									3.14			
Steam distillation	H_2_O	100	ns	60	ns	ns	Dried	ns	EO	NS	Anti-hyperlipidemia	ns	[37]
Soxhlet extraction	EtOH	BTB	ns	360–480	ns	ns	Dried	ns	Extract	16.38	Antioxidant, antibacterial	Functional food	[9]
Steam distillation	H_2_O	100	ns	240	ns	ns	Grinding	Powder	EO	0.57			
Hydro-distillation	H_2_O	100	ns	180	ns	ns	ns	ns	EO	0.31	ns	ns	[35]
Hydro-distillation	H_2_O	ns	ns	180	ns	ns	ns	ns	EO	0.36	ns	ns	[4]
Steam distillation		100								0.04			
**New Extraction Techniques**													
MAE	H_2_O	25	ns	60	ns	ns	ns	ns	EO	1.19 (*v*/*w*)	Protection hepatocellular I/R damage	Food additives as flavoring	[18]
UAE	EtOH 80%	ns	ns	30	100	ns	Grinding	Powder	EO	NS	Anti-hyperlipidemia	ns	[37]
OAHD	H_2_O	ns	ns	50	ns	ns	ns	ns	EO	0.05	ns	ns	[4]
SLME	H_2_O								EO	0.21			
SFME	-								EO	0.17			
UAE	EtOH 80%	80	ns	180	ns	ns	ns	ns	Alkaloids	5.66	Inhibition lipid accumulation	Pharmaceutical industry	[17]

Abbreviations: UAE: ultrasound-assisted extraction; OAHD: ohmic assisted hydro-distillation; SLME: solventless microwaved extraction; SFME: solvent-free microwave extraction; S: solvent; T: temperature; t: time; RT: room temperature; ns: not specified; EO: essential oil; TPC: total phenolic compounds; TFC: total flavonoid content; MeOH: methanol; EtOH: ethanol; EtOAc: ethyl acetate; I/R ischemia/reperfusion; DW: dry weight; EAG: gallic acid equivalents; EC: catechin. BTB: below the boiling point.

**Table 3:** Evaluation of the advantages and limitations of conventional and modern extraction techniques.

EM	Advantages	Limitations	Ref
**Conventional Methods**			
Mechanochemical	- Increasing the extraction rate - Reduction in solvent use and extraction time	- Modification of crystal structure, physical, and chemical properties	[34]
Reflux extraction	- High yield shorter extraction time - Less solvent use	- Loss of heat-sensitive compounds	[42]
Hydro-distillation	- Increase the extraction yield - Centrifugation and filtration not required	- Long extraction time - Loss of heat-sensitive compounds	[34,43]
Percolation	- Shorter extraction time - Thermolabile compound extraction - Shorter extraction time	- Very long extraction time - Higher solvent consumption - Complex protocol	[44]
Soxhlet	- Automatic continuous process - Increase extraction yield - No filtration or centrifugation needed	- Loss of heat-sensitive compounds - Higher solvent consumption - Long extraction time	[44,45]
Steam distillation	- Safe and easy protocol - Higher purity	- Long extraction time - High energy consumption	[46,47]
**New Extraction Methods**			
MAE	- Lower solvent consumption - Higher extraction yield - Better selectivity, and reproducibility	- Loss of heat-sensitive compounds - High energy consumption - Safety precautions	[48]
UAE	- Higher extraction yield - Short extraction time - Lower solvent consumption - Preservation of thermosensitive compounds	- Cost of equipment - Limitation on compound extraction - Impact on functional properties	[49]
OAHD	- Shorter extraction time - Higher extraction yield	- Cost of equipment - Loss of sensitive compounds - Safety precautions	[50]
SLME	- Lower solvent consumption - Higher extraction yield - Shorter time	- Loss of heat-sensitive compounds - High energy consumption	[4]
SFME	- No solvent consumption - Higher extraction yield - Shorter time	- Loss of heat-sensitive compounds - High energy consumption	[4]

Abbreviations: MAE: microwave-assisted extraction UAE: ultrasound-assisted extraction; OAHD: ohmic-assisted hydro-distillation; SLME: solventless microwaved extraction; SFME: solvent-free microwave extraction.

## Data Availability

No new data were created or analyzed in this study. Data sharing is not applicable.
